# A novel platform for virus-like particle-display of flaviviral envelope domain III: induction of Dengue and West Nile virus neutralizing antibodies

**DOI:** 10.1186/1743-422X-10-129

**Published:** 2013-04-24

**Authors:** Anthony JS Chua, Cyrielle Vituret, Melvin LC Tan, Gaëlle Gonzalez, Pierre Boulanger, Mah-Lee Ng, Saw-See Hong

**Affiliations:** 1 Flavivirology Laboratory, Department of Microbiology, National University of Singapore, 5 Science Drive 2, Singapore, 117597, Singapore; 2 NUS Graduate School for Integrative Sciences and Engineering, National University of Singapore, 28 Medical Drive, Singapore, 117456, Singapore; 3 University Lyon I & UMS-3444 Biosciences Gerland-Lyon Sud, 50, avenue Tony Garnier, Lyon, 69366, France; 4 Retroviruses and Comparative Pathology, Université Lyon I & INRA UMR-754, 50, avenue Tony Garnier, Lyon Cedex 07, 69366, France; 5 Flavivirology Laboratory, Department of Microbiology, National University Health System, 1E, Kent Ridge Road, Singapore, 119228, Singapore; 6 Institut National de la Santé et de la Recherche Médicale, 101, rue de Tolbiac, Paris, 75013, France

**Keywords:** Flavivirus envelope glycoprotein, Domain III, Retroviral Gag, Virus-like particles (VLPs), Pseudotyping, VLP-display, CD16/FcϵRIγ chimera, Recombinant baculovirus

## Abstract

CD16-RIgE is a chimeric human membrane glycoprotein consisting of the CD16 ectodomain fused to the transmembrane domain and cytoplasmic tail of the gamma chain of the high affinity receptor of IgE (RIgE). Coexpression of CD16-RIgE and HIV-1 Pr55Gag polyprotein precursor (Pr55Gag^HIV^) in insect cells resulted in the incorporation of CD16-RIgE glycoprotein into the envelope of extracellular virus-like particles (VLPs), a phenomenon known as pseudotyping. Taking advantage of this property, we replaced the CD16 ectodomain of CD16-RIgE by the envelope glycoprotein domain III (DIII) of dengue virus serotype 1 (DENV^1^) or West Nile virus Kunjin (WNV^Kun^). The two resulting chimeric proteins, DIII-DENV^1^-RIgE and DIII-WNV^Kun^-RIgE, were addressed to the plasma membrane, exposed at the surface of human and insect cells, and incorporated into extracellular VLPs when coexpressed with Pr55Gag^HIV^ in insect cells. The DIII domains were accessible at the surface of retroviral VLPs, as shown by their reactivity with specific antibodies, and notably antibodies from patient sera. The DIII-RIgE proteins were found to be incorporated in VLPs made of SIV, MLV, or chimeric MLV-HIV Gag precursors, indicating that DIII-RIgE could pseudotype a wide variety of retroviral VLPs. VLP-displayed DIII were capable of inducing specific neutralizing antibodies against DENV and WNV in mice. Although the neutralization response was modest, our data confirmed the capability of DIII to induce a flavivirus neutralization response, and suggested that our VLP-displayed CD16-RIgE-based platform could be developed as a vaccine vector against different flaviviruses and other viral pathogens.

## Introduction

Flaviviruses like dengue virus (DENV) and West Nile virus (WNV) have established strongholds in many parts of tropical and sub-tropical countries world-wide. They are among the most important agents of re-emerging diseases. These viruses are transmitted by mosquito vectors and with global warming, the potential territorial expansion for the spread of these diseases are real. According to WHO (http://www.who.int/mediacentre/factsheets/fs117/en/), DENV affects an estimated 50 to 100 million people each year. To date, there is still no efficient vaccine nor antiviral for human use for either virus. Efficient vaccines are those inducing a robust neutralizing immune response, and the presentation of epitopes inducing virus neutralization activity (NA) is obviously optimal in whole virus vaccines, and in virus-like particles (VLPs) which mimic natural virions [1]. This is best illustrated by the VLP-based strategy against human papilloma virus (HPV), recently approved for human vaccination against HPV infections and HPV-associated cervical lesions cancerous in nature [2,3].

VLPs are highly organized particulate structures, which differ in nature and composition from infectious virions, most importantly by lacking the viral genome. VLPs can be made up solely of viral capsid proteins such as in the case of HPV, as mentioned above, and hepatitis E virus (HEV) [4], or of viral coat proteins in the case of bacteriophage AP205 [5]. VLPs consisting only of viral envelope glycoproteins have been isolated, such as the subviral particles formed by hepatitis B virus (HBV) envelope glycoprotein S, or by chimeric HBV and hepatitis C virus (HCV) envelope proteins [6,7]. In the case of the influenza virus, VLPs produced by recombinant baculovirus-infected insect cells consisted of two membrane-associated proteins, hemagglutinin and matrix protein [8].

VLPs based on retroviruses or lentiviruses, e.g. human immunodeficiency virus (HIV), represent another type of subviral structure. They are membrane-enveloped particles made up of a backbone of viral polyprotein Gag (or Gag precursor; Pr), wrapped by an envelope derived from the host cell plasma membrane. The HIV-1 Gag precursor (Pr55Gag) spontaneously assembles into VLPs, which bud at the plasma membrane of Gag-expressing insect cells, and are structurally similar to immature virions except that they do not contain any viral genetic material [9-15]. During their budding process, the membrane-enveloped viruses and VLPs bring with them glycoproteins of both viral and nonviral origins present in the plasma membrane of producer cells [16]. Substitution of the viral envelope gene by another type of envelope glycoprotein is a phenomenon which is referred to as pseudotyping. Pseudotyping has been described for various enveloped viruses, including retroviruses, lentiviruses, vesicular stomatitis virus (VSV), HCV and baculoviruses [17-22].

The non-replicative nature of VLPs and their structural analogy to natural viruses make them highly attractive candidates for the design of subunit vaccines which carry the immunogenic structures of the virus in the absence of infectious genetic material. In addition, VLPs can induce both humoral and cellular mediated immune responses [23]. Considering that mosquitos are intermediate hosts for DENV and WNV, the use of the baculovirus-insect cell expression system for the production of VLPs pseudotyped by the envelope glycoprotein (E) of DENV and WNV, or by E-derived structural domains, represents a logical approach for the development of VLP-based vaccines against these flaviviruses. The E domain III (DIII) was chosen as our candidate subunit vaccine in view of several reasons. DIII is the putative receptor binding domain in flaviviruses. Antibodies that bind specifically to DIII have been shown to be highly efficient in preventing virus adsorption to cells [24,25]. DIII contains numerous neutralizing epitopes to which binding of specific antibodies can effectively lead to neutralization of the virus. In addition to several other groups, we have also demonstrated that DIII is highly immunogenic. When used as subunit vaccines, DIII is able to induce protective immune responses against flaviviruses in both rodents and non-human primates [26-37].

CD16-RIgE is a chimeric receptor molecule consisting of the ectodomain of CD16 fused to the transmembrane and cytoplasmic domains of FcϵRIγ (abbreviated RIgE in the present study), the gamma polypeptide chain of the high affinity IgE-Fc receptor [38]. Human CD16 (or FcγRIIIa) is one of the low-affinity receptors for IgG Fc and is involved in antibody-dependent cell-mediated cytotoxicity (ADCC). It allows the recognition of IgG-sensitized target cells by CD16-bearing cytotoxic cells (i.e. CD56^dim^ natural killer cells [39], a fraction of monocytes, macrophages and rare T cells), and the activation of these effector cells [38]. When expressed in human cells, the CD16-RIgE glycoprotein is trafficked to the cell surface via the Golgi pathway [38].

In the present study, we designed a strategy of pseudotyping retrovirus-based VLPs consisting of the co-expression of a retroviral Gag polyprotein, forming the VLP scaffold, and CD16-RIgE in insect cells. The polyprotein Pr55Gag^HIV^ in HIV-1-infected human cells is addressed to the late endosomal compartment and plasma membrane, via its N-myristoyl group and a cluster of basic residues located in the matrix domain [9,16,40]. In Sf9 insect cells, the recombinant Pr55Gag^HIV^ is also addressed to the plasma membrane, and efficiently self-assembles into VLPs, which bud and egress from the cell surface [10,13-15,41]. CD16-RIgE was found to be efficiently incorporated into the envelope of extracellular VLPs, and served as the pseudotyping platform. We showed that substitution of the CD16 ectodomain by the DIII domain from DENV or WNV E glycoprotein resulted in a chimeric flaviviral/human fusion protein DIII-RIgE, which was incorporated into extracellular VLPs. The DIII domain was exposed at the surface of the VLP envelope, and VLP-displayed DIII epitope(s) induced DENV and WNV neutralizing antibodies in mice. These results suggested that our VLP-displayed CD16-RIgE-based platform could be applied to the generation of recombinant vaccines against various viral and microbial pathogens.

## Materials and methods

### Cells and viruses

***(i) Insect cells***. *Spodoptera frugiperda* Sf9 cells were maintained as monolayers at 28°C in Grace’s insect medium supplemented with 10% fetal bovine serum (FBS) and antibiotics (Invitrogen). They were infected with recombinant baculovirus at a multiplicity of infection (MOI) ranging from 5 to 10 PFU/cell, as previously described [11,13,14,41-45]. In co-expression experiments, Sf9 cells were infected with two recombinant baculoviruses simultaneously at equal MOI [45-47]. ***(ii) Mammalian cells***. Human embryonic kidney HEK293 cells were obtained from the American Type Culture Collection (ATCC; Manassas, VA), and maintained as monolayers in Dulbecco’s modified Eagle’s medium (DMEM; Invitrogen) supplemented with 10% FBS, penicillin (100 U/mL), and streptomycin (100 mg/mL) at 37°C and 5% CO_2_. Baby hamster kidney BHK21 cells used for plaque assays were maintained in conditions similar to HEK293 cells, in Roswell Park Memorial Institute-1640 medium (RPMI-1640; Sigma-Aldrich). ***(iii) Viruses.*** The Dengue serotype 1 virus used throughout this work was a laboratory-adapted clinical strain isolated in Singapore. It was a kind gift from the Environmental Health Institute (EHI), Singapore. The WNV Kunjin strain (WNV^Kun^) belongs to the MRM 61C strain, a kind gift from the late Professor E.G. Westaway, Monash University, Australia [48]. Both viruses were propagated in *Aedes albopictus* mosquito C6/36 cell line in Leibovitz-15 medium (L-15; Sigma-Aldrich) supplemented with 10% heat-inactivated FBS at 28°C.

### Recombinant baculoviruses

All foreign genes were inserted into the genome of *Autographa californica* MultiCapsid NucleoPolyhedrosis Virus (AcMNPV) under the control of a chimeric AcMNPV-GmNPV polyhedrin promoter, as described in previous studies [12-14,41,44,46,49]. AcMNPV-Pr55Gag^HIV^ expressed the N-myristoylated, full-length Gag polyprotein (Pr55Gag^HIV^) of HIV-1 [12-14]. AcMNPV-Pr57Gag^SIV^, AcMNPV-Pr65Gag^MLV^ and AcMNPV-Pr72Gag^MLV-HIV^ have been described in a previous study [44].

### VLP production and isolation

Culture supernatants of baculovirus-infected, Gag-expressing Sf9 cells were clarified by low-speed centrifugation. VLPs were then recovered using a two-step procedure comprising 20% sucrose cushion ultracentrifugation, followed by ultracentrifugation in linear sucrose-D_2_O gradient [44,47,49]. In brief, (a) VLPs were first recovered by pelleting through a cushion of 20% sucrose in TNE buffer (TNE: 100 mM NaCl, 10 mM Tris–HCl pH 7.4, 1 mM Na_2_EDTA) at 30,000 rpm for 1 h at 15°C in a Kontron TST-55.5 rotor. VLP pellets from step (a) were gently resuspended in PBS (0.20-0.25 mL), and (b) further purified by isopycnic ultracentrifugation in sucrose-D_2_O gradients. Linear gradients (10-mL total volume, 30-50% w:v) were centrifuged for 18 h at 28,000 rpm in a Beckman SW41 rotor. The 50% sucrose solution was made in D_2_O buffered to pH 7.2 with NaOH, and the 30% sucrose solution was made in 10 mM Tris–HCl, pH 7.2, 150 mM NaCl and 5.7 mM Na_2_EDTA. Aliquots of 0.4 mL were collected from the top, and proteins analysed by SDS-PAGE and immunoblotting. Protein concentration in samples was determined by Bradford protein assay (Thermo Fisher Scientific Inc.).

### Determination of VLP titers

Cryoelectron microscopy and scanning electron microscopy have shown that one immature HIV-1 particle (the structural equivalent of VLPs; [15]) contains approximately 5,000 copies of closely packed Pr55Gag protein [50-52]. The HIV-1 mature capsid is a fullerene-like conical structure composed of approximately 250 hexamers and 12 pentamers of the viral CAp24 protein [53], generated by proteolytic cleavage of Pr55Gag molecules. The fullerene core accounts for 1,560 copies of CAp24 per core, a theoretical number which is consistent with the value of 1,500 copies of CAp24 protein per mature core, determined by biochemical methods [52,54]. This implied that less than half of the Pr55Gag molecules are used to form mature cores made of CAp24 proteins [50-52]. The protein concentration in VLPs was estimated by SDS-PAGE analysis of VLP samples, by comparing the intensity of the Pr55Gag signal after Coomassie blue staining with a range of bovine serum albumin (BSA) samples of known concentrations co-electrophoresed in the same gel. Taking into account the value of 5,000 Pr55Gag copies per VLP, we calculated that a concentration of 1 mg/mL of Pr55Gag protein corresponded to a titer of ~ 2 × 10^12^ VLPs per mL.

### Construction of AcMNPV expressing chimeric human glycoprotein CD16-RIgE

The plasmid pcDNA3.1/FcγRIIIa/FcϵRIγ, expressing the chimeric human construct abbreviated CD16-RIgE in the present study was obtained from Henri Vié and Béatrice Clémenceau (INSERM U-609, Nantes; [38]). CD16-RIgE consisted of the fusion of the ectodomain of FcγRIIIa (or CD16), the human low-affinity receptor for IgG Fc (involved in antibody-dependent cell-mediated cytotoxicity; ADCC), to the transmembrane and cytoplasmic domains of the gamma polypeptide of the human high affinity receptor for IgE, FcϵRIγ. In terms of functionality, the CD16 ectodomain was responsible for the recognition of IgG Fc, whereas FcϵRIγ transduced the intracellular signals [38]. The cDNA encoding CD16-RIgE was rescued using conventional overlapping PCR, and inserted into the *Nhe*I and *Hind*III sites of the pBlueBac4.5 vector genome (Invitrogen). Recombinant AcMNPV-CD16-RIgE were isolated using beta-galactosidase staining of positive Sf9 cells and blue plaque selection.

### Construction of recombinant AcMNPV expressing flaviviral/human chimeric protein DIII-RIgE

**Figure 1:**
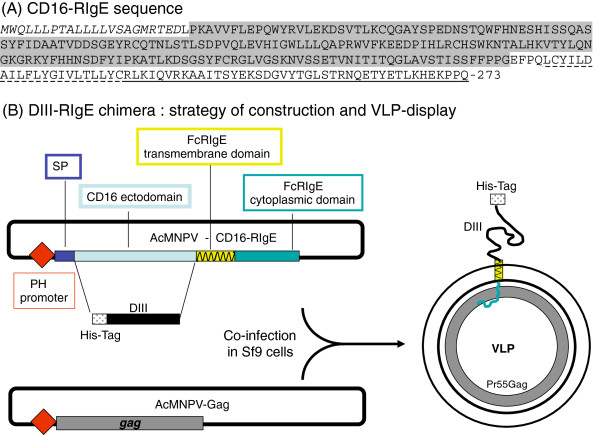
**VLP-display of flavivirus antigenic DIII domain.** (**A**), Amino acid sequence of the CD16-RIgE cloning platform. The CD16-RIgE is a chimeric molecule composed of the extracellular domain of CD16, and transmembrane (TM) domain and cytoplasmic tail (CT) of the gamma chain of the human high affinity IgE receptor (FcϵRIg), abbreviated RIgE in the present study. Symbols for the different domains of the amino acid (aa) sequence are the following: italics, signal peptide (aa residues 1-22); standard capitals, ectodomain of CD16 (aa 23-209); underlined with dotted line, transmembrane domain (aa 210-231); underlined with solid line, cytoplasmic domain (aa 232-273); shaded residues, deletion (aa 23-206) of the CD16 ectodomain, replaced by the flavivirus envelope DIII domain. (**B**), Strategy of construction of the DIII-RIgE chimera (*left*), and of VLP-display (*right*), by co-expression with HIV-1 Pr55Gag in Sf9 cells doubly infected with baculovirus recombinants AcMPV-DIII-RIgE and AcMNPV-Pr55Gag^HIV^.

The DIII domains of Dengue virus serotype 1 (DENV^1^) and West Nile Virus Kunjin (WNV^Kun^) have been cloned into the pET28a vector (Novagen), and expressed in bacterial cells [28,29,31,55,56]. Both clones contained an oligo-histidine sequence (His_6_-tag) at their N-termini. The CD16 ectodomain was deleted from the pcDNA3.1/CD16-RIgE vector, by double cleavage at the *Bgl* II and *Eco*R I sites, and substituted with the cDNA fragment of His_6_-tagged DIII domain from the two flavivirus selected as prototypes: DENV^1^ and WNV^Kun^. The final constructs were abbreviated pcDNA3.1-DIII-DENV^1^-RIgE and pcDNA3.1-DIII-WNV^Kun^-RIgE. Each construct encoded, from the N- to C-terminus, the CD16 signal peptide, His_6_-tag, DIII domain, and RIgE C-terminal transmembrane and cytoplasmic moieties, as depicted in Figure 1. Each construct was then reinserted into the pBlueBac4.5 vector genome, to generate two recombinant baculoviruses AcMNPV-DENV^1^-RIgE and AcMNPV-WNV^Kun^-RIgE.

### Gel electrophoresis, membrane transfer and antibodies

Polyacrylamide gel electrophoresis of SDS-denatured protein samples (SDS-PAGE), and immunoblot analysis have been described in detail in previous studies [44,47,49]. Briefly, samples were denatured in SDS/beta-mercaptoethanol-containing loading buffer at 100°C for 2 min, and proteins electrophoresed in SDS-denaturing 10% polyacrylamide gel and electrically transferred to nitrocellulose membrane (Hybond™-C-extra; GE Healthcare Life Sciences). Blots were blocked in 5% skimmed milk in Tris-buffered saline (TBS) containing 0.05% Tween-20 (TBS-T), rinsed in TBS-T, then successively incubated with primary antibody and phosphatase- or peroxidase-labeled secondary antibodies. Mouse monoclonal anti-His_6_ tag antibody was purchased from Clontech Laboratories Inc. (6xHis monoclonal antibody), and anti-human CD16 mouse monoclonal antibody DJ130C from Santa Cruz Biotechnology. Anti-HIV-1 Gag rabbit polyclonal antibody (laboratory-made; [44]) was raised in rabbit by injection of the band of Pr55Gag protein extracted from SDS-gel. The anti-Pr55Gag^HIV^ serum was found to efficiently react with Pr57Gag^SIV^[44]. Rabbit polyclonal antibody to MLV-GagCAp30 protein was purchased from antibodies-online Inc. When necessary, proteins were detected on blots by reaction with peroxidase-labeled antibody and SuperSignal® West Pico chemiluminescence substrate (Thermo Fisher Scientific Inc.). Luminograms were visualized using the Fusion X7 imaging system with the Bio1D software (Vuilbert-Lourmat, Marne-la-Vallée, France). Apparent molecular weights were estimated by comparison with prestained protein markers (Precison Plus Protein™ Standards, Dual Color; Bio-Rad Laboratories, Inc.).

### ELISA

The accessibilty of recombinant DIII proteins at the surface of VLPs was evaluated by their reactivity with specific anti-flavivirus antibodies in standard ELISA procedure. In brief, aliquots (100 μL) of VLP suspension (50 μg/mL; 10^11^ VLPs/mL) in coating buffer (0.1 M NaHCO_3_, pH 9.6) were incubated overnight at 4°C in 96-well Maxisorp plates (NUNC). The plates were blocked at 37°C for 30 min using serum diluent made up of PBS with 0.1% Tween 20 (PBS-T) and 5% skimmed milk. For DIII-DENV^1^, patients sera positive for DENV infection, used under ethical approval, was diluted 100 times with serum diluent. For DIII-WNV^Kun^, two antibodies were used: mouse monoclonal antibody 3.67G, raised against the Kunjin envelope protein (Chemicon International, Inc.), recognizing a DIII epitope [57]; anti-WNV-DIII rabbit polyclonal antibody, a kind gift from S. Lecollinet, Ecole Nationale Vétérinaire d’Alfort, Maisons-Alfort, France [58]. Reagents were added to wells in the following order: diluted human antisera or primary animal antibodies, diluted (1:250) secondary anti-human, anti-rabbit or anti-mouse IgG-biotin conjugate (Chemicon International, Inc.), diluted (1:5,000) streptavidin-HRP conjugate (Chemicon International, Inc.), TMB One substrate solution (Promega Corporation), and 0.5 M sulfuric acid stop-solution. All wells were washed with PBS-T thrice before the addition of each reagent, except before the stop solution was added. Plates were also incubated for 1 h at 37°C after the addition of each reagent. Absorbance was finally measured at 450 nm.

### Immunofluorescence (IF) microscopy

Baculovirus-infected Sf9 cell monolayers were harvested at 48 h post infection (hpi), fixed with 3% paraformaldehyde in phosphate buffered saline (PBS) and, when required, permeabilized with 0.2% Triton X-100 in PBS. Cells were blocked with 3% BSA in PBS (PBS-BSA), and His-tagged DIII detected at the surface of nonpermeabilized cells by reaction with monoclonal anti-His_6_ tag antibody (1:1,000 in PBS-BSA) and Alexa Fluor® 488-labeled goat anti-mouse IgG antibody (Life Technologies Corp.). For double labeling of DIII and Gag proteins, Sf9 cells were permeabilized and reacted with rabbit anti-Gag antibody (1:1,000 in PBS-BSA) and Alexa Fluor® 594-labeled goat anti-rabbit IgG (Life Technologies Corp.). HEK293 cell samples were labeled using Image-iT™ LIVE Plasma Membrane and Nuclear Labeling Kit (Invitrogen), which provides red-fluorescent Alexa Fluor® 594 wheat germ agglutinin (WGA) and blue-fluorescent Hoechst-33342 dye for plasma membrane and nucleus staining, respectively. For confocal microscopy, samples were analyzed using a Nikon A1R fast laser scanning confocal microscope.

### Electron microscopy (EM) and immuno-EM

VLP specimens were processed and observed under EM as previously described [17]. In brief, VLPs were diluted in 20 μL 0.14 M NaCl, 0.05 M Tris–HCl buffer, pH 8.2 (Tris-buffered saline; TBS) and adsorbed onto carbon-coated formvar membrane on grids. The grids were incubated with primary antibody (monoclonal anti-His_6_ tag antibody) at a dilution of 1:50 in TBS for 1 h at room temperature (RT). After rinsing with TBS, the grids were post-incubated with 10-nm colloidal gold-tagged goat anti-mouse IgG antibody (British Biocell International Ltd, UK; diluted to 1:50 in TBS) for 30 min at RT. After rinsing with TBS, the specimens were negatively stained with 1% sodium phosphotungstate in H_2_O for 1 min at RT, rinsed again with TBS, and examined under a JEM 1400 JEOL electron microscope equiped with an Orius-Gatan digitalised camera (Gatan, France).

### Mice immunization

The immunization regime was inspired from the protocol previously described to obtain neutralization antibodies in mice immunized with inactivated flavivirus particles used as immunogens [28,29]. In our protocol published earlier, BALB/C mice were injected intraperitoneally with 100 μL-aliquot per animal of virus stock purified to 10^7^ PFU/mL with complete Freund’s adjuvant. This was repeated at weeks 3, 5 and 7 with incomplete Freund’s adjuvant, and animals were bled at week 8. Considering that the infectivity index (IP:VP ratio) of flaviviruses varies between 1:100 and 1:1,000 [59], we estimated that a 100 μL-dose containing 10^6^ infectious particles (IP) corresponded to 10^8^ to 10^9^ physical virus particles (VP). Knowing that the outer glycoprotein shell of a mature flavivirus particle is formed by 30 rafts of three homodimers of the viral surface E protein, viz. 180 copies of E monomer per virion [60], we calculated a range of values of 1.8 × 10^10^ to 1.8 × 10^11^ copies of E monomer per dose. The protocol used in the present study for immunization with DIII-RIgE-pseudotyped VLPs was based on the following parameters: (i) The above-mentioned estimation of the number of 10^8^-10^9^ VP and 1.8 × 10^10^-1.8 × 10^11^ copies of E monomer per dose; (ii) our calculation that 1 μg total Gag protein (full-length Pr55Gag + major cleavage product Pr41Gag) corresponded to 2 × 10^9^ VLP; (iii) the average number of 300 copies of DIII-RIgE copies per VLP (360 molecules of DIII-DENV^1^-RIgE and 240 molecules of DIII-WNV^Kun^-RIgE per VLP, as detailed below in the Results section). (iv) The immunization route in the work presented here took into account ethical considerations: VLP samples mixed with complete or incomplete Freund’s adjuvant were injected into animals via the subcutaneous route, instead of the peritoneal route previously used [28,29]. Three series of five 11 week-old mice were injected with 12-15 μg total Gag protein (viz. 2.4 × 10^10^ to 3 × 10^10^ VLPs per dose, accounting for a range of 7 × 10^12^ to 9 × 10^12^ DIII copies per dose) with complete Freund’s adjuvant, in a total volume of 40-50 μL. The DIII doses administered subcutaneously with VLPs were therefore 50-fold higher than the dose of E-monomer protein used in our previous immunization protocol with inactivated virions injected intraperitoneally. One series of mice received VLP-DENV^1^, another received VLP-WNV^Kun^, and the third series consisted of mice injected with nonpseudotyped VLPs (VLP-0), to serve as negative control. Injections were repeated at weeks 3, 5 and 7 with the same doses, along with incomplete Freund’s adjuvant, and animals bled at week 8. Sera were probed for anti-DENV^1^ and anti-WNV^Kun^ antibodies using ELISA, and for virus neutralizing antibodies using plaque reduction neutralization test.

### Assays for humoral response in mice immunized with VLPs

***(i) ELISA*.** The ELISA protocol utilized here was similar to that used to determine the binding of patient antisera to pseudotyped VLPs. (i) Five hundred PFU of live virus was coated onto well bottoms. (ii) Mouse sera raised against DIII-pseudotyped VLPs or VLP-0 negative control were used as primary antibodies. (iii) Anti-mouse IgG-biotin conjugate (Chemicon Inc.) was used as secondary antibodies.

***(ii) Plaque reduction neutralization test (PRNT).*** The occurrence of flavivirus neutralizing antibodies in mouse sera was assayed using PRNT [28,29]. Prior to PRNT, all sera were inactivated at 56°C for 30 min. Heat-inactivated sera were diluted in RPMI containing 2% FBS (virus diluent) at 1:10 ratio, and incubated with 500 PFU of virus at 37°C for 1 h. The virus-antibody mixtures were transferred in triplicates onto confluent BHK cell monolayers, and incubated at 37°C for 1 h. The cell monolayers were then washed with virus diluent, and overlaid with overlay medium. Depending on the virus type, plaques were visualized by staining the monolayer with 0.5% crystal violet in a 25% formaldehyde solution at three (WNV^Kun^) or four (DENV^1^) days post-infection.

### Ethics statement

All patients positive for DENV^1^ infection provided written informed consent for the use of their sera, which were used under ethical approval by the Domain Specific Review Boards of Singapore (DSRB, Ref. B/05/013). Animals used in this study were handled at Eurogentec SA (Belgium), in compliance with the following association’s requirements: the Federation of European Laboratory Animal Science Associations (FELASA), the UK Home Office Animals Scientific Procedures Act, and the USA National Institutes of Health Approved Supplier (Animal Welfare Assurance #A5337-01). FELASA (http://www.felasa.eu) is a consortium of European Community government animal ethics committees dedicated to the ethical use of research animals across all EU countries. The immunization work was performed at Eurogentec SA, and our immunization protocol approved by the Ethics Committee of the Centre d'Economie Rurale (CER Groupe, Marloie, Belgium). Our animal protocol received the permit number FR10277-10278-10279.

## Results

### Incorporation of CD16-RIgE into VLPs produced by insect cells

**Figure 2:**
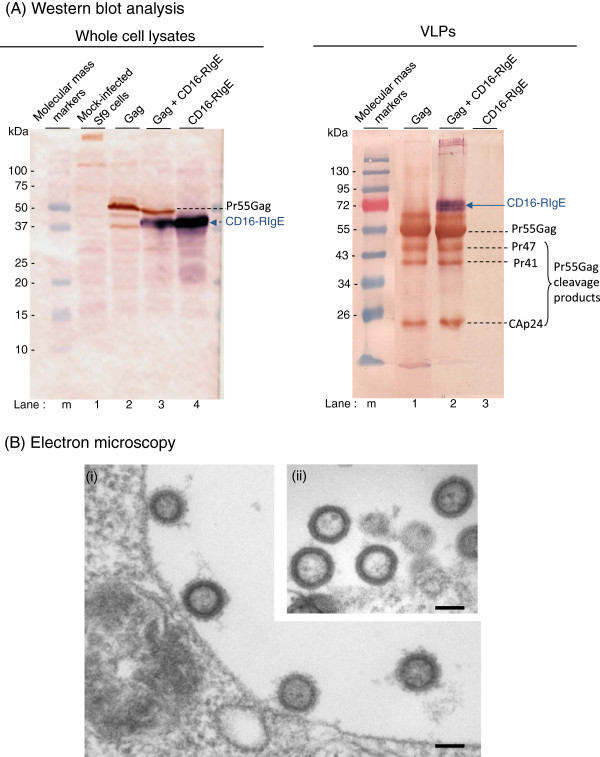
**Pseudotyping of VLPs with CD16-RIgE protein.** (**A**), *Western blot analysis of CD16-RIgE-expressing cells and CD16-RIgE-pseudotyped VLPs.* Left panel: *whole cell lysates.* Sf9 cells were mock-infected (lane **1**), infected with AcMNPV-Pr55Gag^HIV^ (lane **2**), coinfected with AcMNPV-Pr55Gag^HIV^ and AcMPV-CD16-RIgE at equal MOI each (10 pfu/cell; lane **3**), or infected with AcMPV-CD16-RIgE (lane **4**). Lysates of cells harvested at 48 hpi were analysed by SDS-PAGE and immunoblotting, using a dual color detection: blots were reacted with anti-Gag rabbit polyclonal antibody (Ab) and peroxidase-labeled anti-rabbit IgG Ab, followed by mouse monoclonal anti-CD16 Ab and phosphatase-labeled anti-mouse IgG Ab. The Pr55Gag^HIV^ polyprotein precursor shows as a sepia red-colored band at 55 kDa (Pr55Gag), and the chimeric CD16-RIgE protein as a sharp purple blue band at 37 kDa. Lane **m**, molecular weight markers. **Right panel:*extracellular VLPs***. VLPs isolated from the culture medium of cells expressing Pr55Gag^HIV^ alone (lane **1**), coexpressing Pr55Gag^HIV^ and CD16-RIgE (lane **2**), and the corresponding samples from CD16-RIgE-expressing cells (no VLP control; lane **3**) were analysed by SDS-PAGE and immunoblotting, as above. The Pr55Gag cleavage products (Pr47, Pr41 and CAp24 [9-15]) are indicated by dotted lines, and the chimeric CD16-RIgE protein, migrating as a blurred band at 72-75 kDa, by a blue arrow. (**B**), ***Electron microscopy (EM) of ultrathin sections of VLP-producing cells***. (**i**), ***Double infection***. Sf9 cells were coinfected with two recombinant baculoviruses AcMNPV-Pr55Gag^HIV^ and AcMNPV-CD16-RIgE, and processed for EM at 48 hpi. **(ii, inset),*Control single infection***. Sf9 cells infected with AcMNPV-Pr55Gag^HIV^ alone produced nonpseudotyped VLP. Note the difference between the fuzzy (i) and smooth (ii) envelopes of VLPs.

This experiment was designed to verify whether in insect cells the two recombinant proteins, CD16-RIgE and Pr55Gag^HIV^, would behave as in mammalian cells, co-localize in the same microdomains of the plasma membrane, and eventually be co-incorporated in budding VLPs. The cDNA for CD16-RIgE was cloned into the genome of baculovirus AcMNPV to generate recombinant AcMNPV-CD16-RIgE (Figure 1). Sf9 cells were infected with AcMNPV-CD16-RIgE or AcMNPV-Pr55Gag^HIV^ alone, or coinfected with AcMNPV-Pr55Gag^HIV^ and AcMNPV-CD16-RIgE. Cells were harvested at 48 hpi, and whole cell lysates and extracellular VLPs, isolated as described in the Materials and Methods section, were probed for CD16-RIgE and Pr55Gag^HIV^ proteins, using SDS-PAGE and Western blot analysis. The intracellular anti-CD16 reacting protein was detected as a major band of 37 kDa in apparent molecular weight (Figure 2A, left panel), whereas it was visible as a blurred band at 72-75 kDa in VLPs (Figure 2A, right panel). The difference in the apparent molecular weights suggested that the CD16-RIgE protein incorporated into VLPs was in a highly glycosylated form. The fact that VLPs were purified by two ultracentrifugation steps, velocity ultracentrifugation through a sucrose cushion, followed by isopycnic ultracentrifugation in a sucrose-D_2_O preformed gradient, and that DIII-RIgE remained associated with Gag polyprotein throughout the purification procedure suggested that retroviral VLPs were pseudotyped with the human glycoprotein CD16-RIgE.

Sf9 cells coinfected with AcMNPV-Pr55Gag^HIV^ and AcMNPV-CD16-RIgE, and control single AcMNPV-Pr55Gag^HIV^-infected Sf9 cells harvested at 48 hpi were processed for electron microscopy (EM). VLPs budding at the plasma membrane of coinfected cells showed a fuzzy aspect of their surface (Figure 2B, i), contrasting with the smooth surface of control, nonpseudotyped VLPs produced by AcMNPV-Pr55Gag^HIV^-infected cells (Figure 2B, ii). This result, which confirmed the immunological analysis of VLPs shown in Figure 2A, implied that CD16-RIgE and Gag were associated as pseudotyped VLPs, and incited us to use CD16-RIgE as a platform for the insertion of flaviviral antigens.

### Expression of fusion proteins DIII-DENV^1^-RIgE and DIII-WNV^Kun^-RIgE in human and insect cells

**Figure 3:**
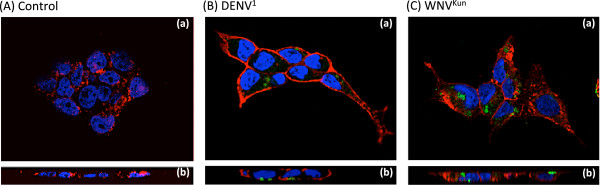
**Confocal immunofluorescence (IF) microscopy of human cells expressing DIII-RIgE fusion proteins.** HEK293 cells were transfected with pcDNA3.1 plasmid expressing DIII-DENV^1^-RIgE or DIII-WNV^Kun^-RIgE, and analysed by fluorescence imaging at 48 h posttransfection without cell permeabilization. Plasma membrane was stained with red-fluorescent WGA, nuclei with blue-fluorescent Hoechst 33342, and DIII-RIgE fusion proteins immunodetected with monoclonal anti-His_6_ tag antibody and Alexa Fluor® 488-labeled goat anti-mouse IgG antibody. (**A**), Control cells transfected with empty pcDNA3.1 plasmid. (**B**), Cells transfected with pcDNA3.1-DIII-DENV^1^-RIgE plasmid. (**C**), Cells transfected with pcDNA3.1-DIII-WNV^Kun^-RIgE plasmid. Panels (**a**) show views from the apical pole; panels (**b**) show the corresponding sagittal cell plane reconstructed from Z-stack images. Note that DIII-DENV^1^-RIgE and DIII-WNV^Kun^-RIgE proteins were accessible at the surface of nonpermeabilized cells, and that they localized in membrane microdomains different from WGA-reacting regions.

Our antigenic constructs included the structural domain DIII of the envelope glycoprotein from two flaviviruses used as prototypes, DENV serotype 1 (DENV^1^) and West Nile virus Kunjin (WNV^Kun^). The rationale for the choice of this domain was the finding that DIII was capable of inducing a robust antiviral response and virus-specific neutralizing antibodies [26-36,56]. The CD16 ectodomain was deleted from the pcDNA3.1/CD16-RIgE vector, and replaced by the coding sequences of His_6_-tagged DIII-DENV^1^ or DIII-WNV^Kun^ (refer to Figure 1). When expressed in human cell line HEK293 using plasmid vector pcDNA3.1, the fusion constructs DIII-DENV^1^-RIgE and DIII-WNV^Kun^-RIgE were found to react with anti-His_6_ tag antibody (immunolabelled with green Alexa Fluor 488-conjugated secondary antibody) in immunofluorescence (IF) confocal microscopy of nonpermeabilized cells, and localized at the plasma membrane, stained with red-fluorescent WGA (Figure 3). This indicated that DIII-DENV^1^-RIgE and DIII-WNV^Kun^-RIgE were addressed to the plasma membrane of HEK293 cells, like parental CD16-RIgE, and that the two DIII domains were exposed at the cell surface, as ectodomains of DIII-RIgE fusion proteins.

**Figure 4:**
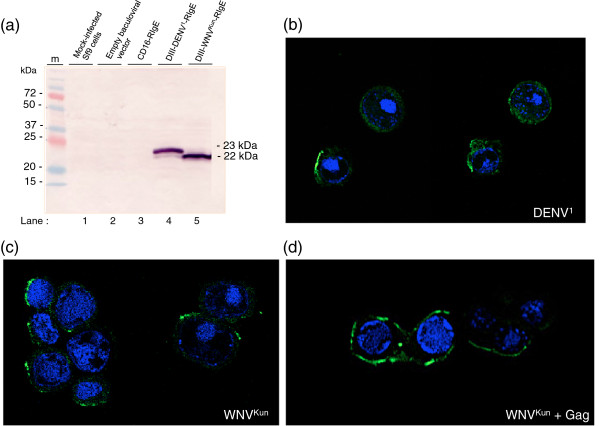
**Expression and localization of DIII-RIgE fusion proteins in insect cells.** (**a**), ***Western blot analysis***. Sf9 were mock-infected (control lane 1), or infected with parental baculovirus vector (control lane 2), recombinant AcMNPV expressing CD16-RIgE (control lane 3), DIII-DENV^1^-RIgE (lane 4), or DIII-WNV^Kun^-RIgE (lane 5). Cells were harvested at 48 hpi and cell lysates analysed by SDS-PAGE and Western blotting. The 22-23 kDa bands of the envelope DIII-RIgE fusion proteins were detected using monoclonal anti-His_6_ tag antibody and complementary phosphatase-labeled antibody. (**b-d**), ***Confocal IF microscopy***. Sf9 cells were infected with AcMNPV expressing DIII-DENV^1^-RIgE (**b**), DIII-WNV^Kun^-RIgE **(c)**, or coinfected with AcMNPV-DIII-WNV^Kun^-RIgE and AcMNPV-Pr55Gag^HIV^ (**d**). Cells were harvested at 48 hpi and reacted with monoclonal anti-His_6_ tag antibody and Alexa Fluor^®^ 488-labeled goat anti-mouse IgG antibody, without cell permeabilization. Nuclei were stained with blue-fluorescent DAPI. Note that the fluorescent signal of the anti-His_6_ tag localized at the cell surface and outlined the cell contour.

Baculovirus vectors were then constructed to express each chimeric protein, DIII-DENV^1^-RIgE or DIII-WNV^Kun^-RIgE alone, or in coexpression with recombinant Pr55Gag^HIV^. The two clones showed a similarly high level of expression of recombinant proteins in Sf9 cells (Figure 4a). They migrated with an apparent molecular mass of 22-23 kDa, a value which was consistent with their amino acid sequences: DIII-DENV^1^-RIgE comprised of 194 residues including the His_6_ tag (6 residues), DIII domain (123 residues), 2 leftover residues from the deleted CD16 ectodomain, and the transmembrane and cytoplasmic domains of FcϵRIγ (63 residues). In IF confocal microscopy, the N-terminal His_6_ tag of DIII-DENV^1^-RIgE and DIII-WNV^Kun^-RIgE chimeric proteins was found to react with anti-His_6_ tag antibody at the cell surface of nonpermeabilized Sf9 cells (Figure 4b, c), indicating that the DIII domain was exposed at the surface of Sf9 cells, as in HEK293 cells.

**Figure 5:**
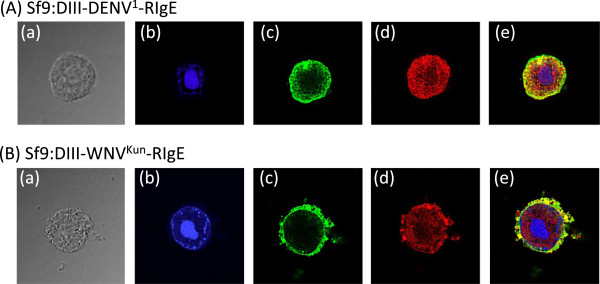
**Colocalization of Pr55Gag**^**HIV **^**and DIII-RIgE in membrane-permeabilized insect cells.** Sf9 cells were coinfected with AcMNPV-Pr55Gag^HIV^ and (**A**) AcMNPV-DIII-DENV^1^-RIgE, or (**B**) AcMNPV-DIII-WNV^Kun^-RIgE, and harvested at 48 hpi. Cells were permeabilized and reacted with monoclonal anti-His_6_ tag antibody and Alexa Fluor® 488-labeled goat anti-mouse IgG antibody, and rabbit polyclonal anti-Gag antibody and Alexa Fluor® 594-labeled anti-rabbit IgG antibody. (**a**), phase contrast; (**b**), DAPI staining, (**c**), Alexa Fluor® 488 channel; (**d**), Alexa Fluor® 594 channel; (**e**), merged fluorescence images. All images were visualized at 100X magnification.

Coexpression with Pr55Gag^HIV^, as detailed below, did not significantly change the confocal IF pattern of membrane-display of DIII-DENV^1^-RIgE or DIII-WNV^Kun^-RIgE (Figure 4d). After cell permeabilization, to give anti-Gag antibody access to intracellular Gag polyprotein, double IF staining showed the co-localisation of Pr55Gag^HIV^ and DIII-DENV^1^-RIgE or DIII-WNV^Kun^-RIgE at the plasma membrane of co-expressing Sf9 cells (Figure 5).

### Incorporation of the DIII-RIgE fusion proteins into VLPs

**Figure 6:**
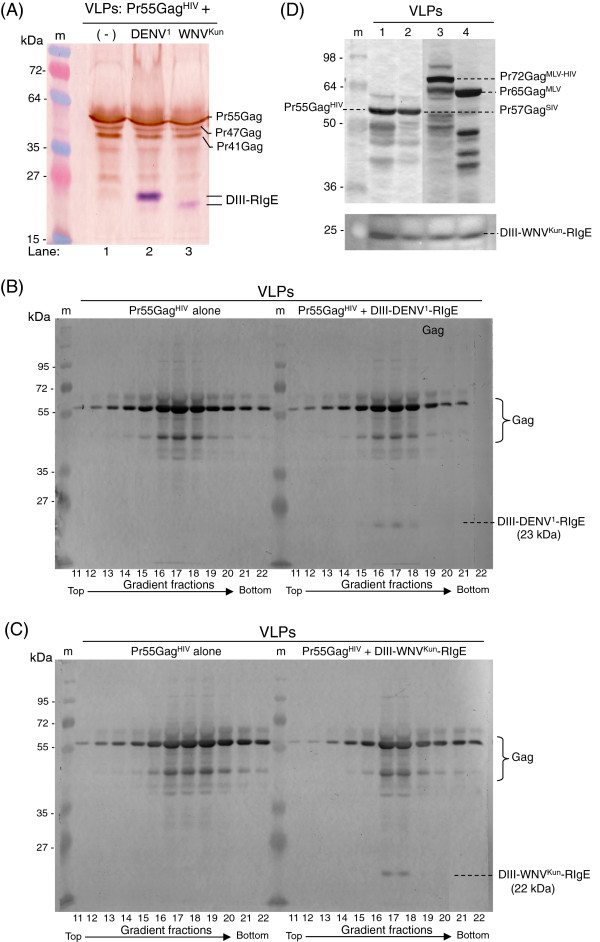
**Protein analysis of DIII-pseudotyped VLPs.** (**A-C**), VLPs were purified from the culture medium of Sf9 cells infected with AcMNPV-Pr55Gag^HIV^ or coinfected with AcMNPV-Pr55Gag^HIV^ and AcMNPV-DIII-DENV^1^-RIgE or AcMNPV-DIII-WNV^Kun^-RIgE. (**A**), VLPs from Pr55Gag^HIV^-expressing cells (lane **1**), from Pr55Gag^HIV^ + DIII-DENV^1^-RIgE coexpressing cells (lane **2**), or from Pr55Gag^HIV^ + DIII-WNV^Kun^-RIgE coexpressing cells (lane **3**) were isolated by centrifugation through a sucrose cushion. VLP samples were analyzed by SDS-PAGE and Western blotting. Dual color was obtained by reaction of the blot with anti-Pr55Gag rabbit antibody and peroxidase-labeled anti-rabbit IgG antibody, which revealed the Pr55Gag^HIV^ polyprotein and its major cleavage products (Pr47Gag^HIV^ and Pr41Gag^HIV^) in sepia red; this was followed by a second reaction with monoclonal anti-His_6_ tag antibody and phosphatase-labeled anti-mouse IgG, which revealed the DIII-RIgE fusion proteins in purple blue. Lane **m**, molecular weight markers. (**B, C**), VLP pellets in (A) from cells coexpressing DIII-RIgE and Pr55Gag^HIV^ were further purified by density ultracentrifugation in sucrose-D_2_O isopycnic gradient, and analyzed by SDS-PAGE and Western blotting, as above. (**B**), DIII-DENV^1^-RIgE; (**C**), DIII-WNV^Kun^-RIgE. (**D**), VLPs were isolated from cells coexpressing DIII-WNV^Kun^-RIgE and (lane **1**) human virus Pr55Gag^HIV^, (lane **2**) simian virus Pr57Gag^SIV^, (lane **3**) chimeric murine-human virus Pr72Gag^MLV-HIV^, or (lane **4**) murine virus Pr65Gag^MLV^. VLPs were analyzed by SDS-PAGE and Western blotting. Blots were reacted with rabbit anti-Gag antibodies and mouse anti-His_6_ tag monoclonal antibody, followed by peroxidase-labeled anti-rabbit IgG and anti-mouse IgG antibodies, then revealed by chemiluminescence substrate. Due to differences in the intensity of immunoreactivity of Gag proteins with polyclonal antibodies, compared to that of DIII-WNV^Kun^-RIgE with monoclonal antibody, the blot was subjected to different exposure times: the luminogram showing the DIII-WNV^Kun^-RIgE protein (lower panel) corresponds to a longer exposure.

In order to test the capacity of each fusion construct to be incorporated into VLPs, Sf9 cells were coinfected pairwise with two baculoviruses, (i) AcMNPV-Pr55Gag^HIV^, which provided the common structural platform, and (ii) AcMNPV-DIII-DENV^1^-RIgE or AcMNPV-DIII-WNV^Kun^-RIgE, which provided the surface protein specific of each VLP pseudotype. Extracellular VLPs were first isolated from the culture medium at 48 hpi, using ultracentrifugation through a 20% sucrose cushion [61]. The DIII signal was found in the VLP pellet, associated with the Gag signal (Figure 6A). VLP pellet was then resuspended in PBS and further purified by isopycnic ultracentrifugation in preformed sucrose-D_2_O gradient. The Gag polyprotein and the His_6_-tagged DIII-DENV^1^-RIgE and DIII-WNV^Kun^-RIgE cosedimented in gradient fractions corresponding to the apparent density of VLPs (ρ = 1.15-1.20; Figure 6B, C). In the absence of expression of Pr55Gag^HIV^, the His_6_-tag signal was found in the gradient top fractions containing soluble proteins (not shown). These results indicated that, when coexpressed with Pr55Gag^HIV^, DIII-DENV^1^-RIgE and DIII-WNV^Kun^-RIgE fusion proteins were *bona fide* components of the Gag VLPs, and were not carried over during the VLP isolation process.

### Influence of retroviral Gag species on the VLP incorporation of DIII-RIgE

It has been shown that incorporation of envelope glycoprotein SUgp120 of HIV-1 into the retroviral particle requires specific interactions between the cytoplasmic tail of TMgp41 with the N-terminal region of the matrix domain (MA) of Pr55Gag^HIV^[9,40,62]. Our next experiment was designed to determine the influence of the Gag polyprotein species on the pseudotyping of VLPs by our chimeric DIII-RIgE constructs. To this aim, we used AcMNPV-Pr57Gag^SIV^, AcMNPV-Pr65Gag^MLV^ and AcMNPV-Pr72Gag^MLV-HIV^ as alternatives to Pr55Gag^HIV^ to constitute the inner backbone of VLPs. AcMNPV-Pr57Gag^SIV^ and AcMNPV-Pr65Gag^MLV^ expressed the N-myristoylated, full-length Gag polyproteins of SIVmac251 (Pr57Gag) and MLV (Pr65Gag), respectively. AcMNPV-Pr72Gag^MLV-HIV^ expressed a chimeric Gag polyprotein consisting of the N-myristoylated MA-CA domains of MLV fused to the SP1-NC-p6 domains of HIV-1 Gag [44]. The incorporation of DIII-WNV^Kun^-RIgE was found to occur in VLP^SIV^, VLP^MLV^ and VLP^MLV-HIV^ (Figure 6D), and similar results were obtained with DIII-DENV^1^-RIgE (not shown). This suggested that incorporation of DIII-RIgE into VLPs was not restricted to one single Gag species, and that the use of VLPs made of other Gag polyprotein species could be envisaged.

### Immunological characterization of VLP-DIII pseudotypes

***(i) Quantitative analysis of DIII incorporation by VLPs.*** The DIII-RIgE content of VLPs was quantitatively determined by SDS-PAGE analysis and gel scanning. VLPs were coelectrophoresed in SDS-polyacrylamide gels with a range of BSA samples of defined concentrations. Gels were stained with Coomassie blue. Gag and DIII protein bands were scanned and quantitated by densitometric analysis, using ImageJ program (NIH) and a quantification method cited in [63] and detailed in the website http://lukemiller.org. Both bands of Pr55Gag^HIV^ precursor and its major cleavage product Pr41Gag were taken into account in our calculation of Gag protein content. We found a protein ratio ranging between 15 and 30 ng of DIII-RIgE per μg total Gag protein (i.e. per 2 × 10^9^ VLPs), and calculated a mean number of 360 ± 30 copies (m ± SEM; n = 4) per VLP for DIII-DENV^1^-RIgE, and 240 ± 25 copies (m ± SEM; n = 4) per VLP for DIII-WNV^Kun^-RIgE. Of note, the flavivirus envelope is composed of 30 rafts containing 3 homodimeric E glycoproteins each, viz. 180 copies of E monomer per particle [60,64]. The difference in the VLP envelope incorporation efficiency between DIII-DENV^1^-RIgE and DIII-WNV^Kun^-RIgE, could not be explained by a difference in the level of cellular expression of DIII-WNV^Kun^-RIgE protein, compared to DIII-DENV^1^-RIgE (refer to Figure 4A), but was likely due to the intrinsic property of each individual DIII construct. Whatever the mechanism, we used the average number of 300 copies of DIII-RIgE copies per VLP to calculate the proper VLP doses to be injected in mice immunization protocols, as detailed in the Materials and Methods section.

**Figure 7:**
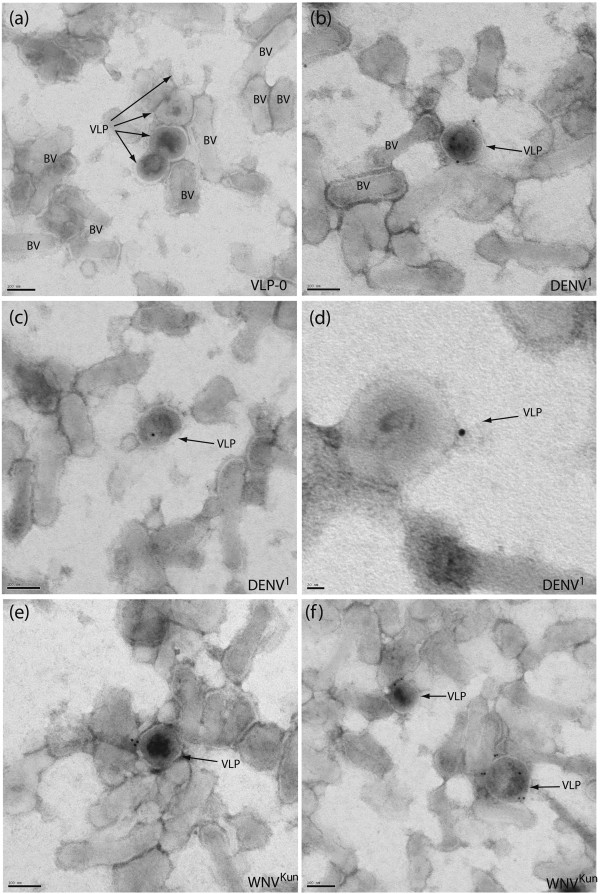
**Immuno-EM analysis of DIII-pseudotyped VLPs.** Extracellular VLPs released from Sf9 cells co-expressing Pr55Gag^HIV^ and DIII-RIgE fusion protein, were isolated by ultracentrifugation through sucrose cushion. Aliquots of VLP samples were adsorbed on EM grids and reacted with monoclonal anti-His_6_ tag antibody, and secondary 10-nm colloidal gold-labeled anti-mouse IgG. (**a**), Control VLPs devoid of DIII (VLP-0); (**b-d**), VLP-displayed DIII-DENV^1^-RIgE; (**e, f**), VLP-displayed DIII-WNV^Kun^-RIgE. Specimens were negatively stained with sodium phosphotungstate. BV, baculovirus vector. Note that all visible gold grains are found to be associated with membrane-enveloped VLPs of 120-130 nm in diameter [12]. Scale bar represents 100 nm in (**a**-**c**) and (**e**-**f**), and 20 nm in (**d**).

***(ii) Immunoelectron microscopy (immuno-EM)***. Accessibility of DIII antigens incorporated in the VLP envelope was first analyzed qualitatively by immunoelectron microscopy, using anti-His_6_ tag antibody to detect the N-terminal His_6_ tag of DIII-DENV^1^-RIgE and DIII-WNV^Kun^-RIgE. The samples consisted of VLPs obtained by ultracentrifugation through sucrose cushion, which were partially purified from the particles of the baculoviral vector. This partial purification through the omission of isopycnic sucrose-D_2_O ultracentrifugation allowed us to assess the specificity of labeling of the different particles. As shown in Figure 7, immunogold labeling was found specifically to associate with VLPs, and not with baculovirions. This demonstrated that DIII-DENV^1^-RIgE and DIII-WNV^Kun^-RIgE were exposed at the surface of Gag VLPs, and immunologically reactive. This further supported our findings in the earlier ultracentrifugation experiments (refer to Figure 6), and provided conclusive visual evidence that DIII-DENV^1^-RIgE and DIII-WNV^Kun^-RIgE fusion proteins were *bona fide* components of Gag VLPs. Of note, costaining of VLPs with anti-Gag antibodies was not possible as the Gag protein is not accessible to antibodies in membrane-enveloped VLPs, whereas the ectodomain of membrane-inserted proteins, such as DIII-RIgE, was exposed to the exterior and fully accessible.

**Figure 8:**
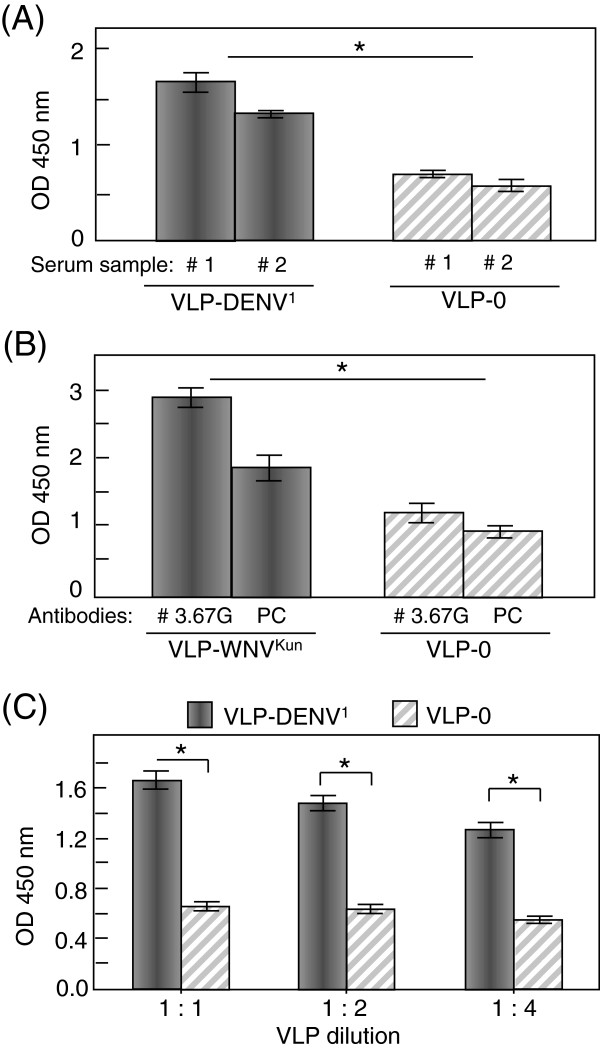
**Immunoreactivity of VLP-displayed DIII.** (**A**), Aliquots of control VLPs devoid of DIII (VLP-0) or VLPs pseudotyped with DIII-DENV^1^-RIgE (VLP-DENV^1^) were coated on plates and probed by ELISA for reactivity towards two different samples of patient sera positive for DENV^1^ infection. (**B**), Aliquots of control VLP-0 or VLPs pseudotyped with DIII-WNV^Kun^-RIgE (VLP-WNV^Kun^) were tested by ELISA for reactivity towards two different types of anti-WNV DIII antibodies, mouse monoclonal 3.67G and rabbit polyclonal (PC) anti-WNV DIII antibodies. (**C**), Dose-dependent reactivity of VLP-DENV^1^ with DENV^1^-specific patient antibodies. Data presented are mean ± SEM, *n* = 4; (*), significantly different at *p* < 0.05 level.

***(iii) ELISA***. In order to confirm the accessibility and specific immunoreactivity of VLP-incorporated DIII-DENV^1^-RIgE and DIII-WNV^Kun^-RIgE, VLP fractions were analyzed by ELISA, using patient sera positive for DENV^1^, and monoclonal and polyclonal antibodies against WNV^Kun^ DIII. The reactivity of VLP-DENV^1^ with specific anti-DENV^1^ antibodies from two different patient sera was significant, compared to the background signals obtained with control VLP devoid of DIII (VLP-0; Figure 8A). Likewise, VLP-WNV^Kun^ reacted with anti-WNV DIII monoclonal and polyclonal antibodies at significant levels (Figure 8B). The immunoreactivity of DIII-DENV^1^-RIgE antigen with anti-DENV^1^ antibodies was found to be VLP dose-dependent (Figure 8C). These results confirmed the structural and antigenic conformation of the DIII antigens at the surface of VLPs, as suggested by the data of IF and immuno-EM using anti-His_6_ tag antibody.

### Immunogenicity of VLP-displayed flaviviral DIII *in vivo*

Three groups of five 11-week old mice were immunized with VLP-displayed (i) DIII-DENV^1^-RIgE, (ii) DIII-WNV^Kun^-RIgE, and (iii) VLP-0, the negative control consisting of nonpseudotyped VLPs.

***(i) Induction of antibodies against live flaviviruses.*** Sera were probed for the presence of antibodies against flavivirus, using ELISA plates coated with live WNV^Kun^ and DENV^1^ virions. In the VLP-DENV^1^-immunized group, sera from 2 out of 5 mice were positive for DENV^1^ virus at all dilutions tested (1:10, 1:50, 1;100, and 1:200), and reacted in a dose-dependent manner (Additional file 1: Figure S1). Similarly, sera from 2 out of 5 mice were positive for WNV^Kun^ virus and reacted in a dose-dependent manner in the VLP-WNV^Kun^-immunized group (Additional file 2: Figure S2). Thus, in mice which responded to the flavivirus DIII antigen at detectable levels, the ELISA showed a moderate antibody response towards the corresponding live virus particles.

**Figure 9:**
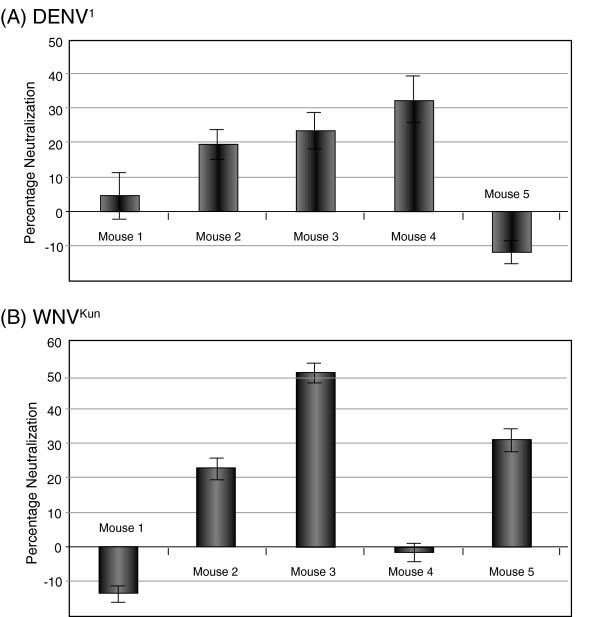
**Induction of flavivirus neutralization antibodies by VLP-displayed DIII antigen.** The occurrence of anti-DENV^1^ or anti-WNV^Kun^ neutralizing antibodies in sera of mice immunized with VLPs displaying the corresponding DIII at their surface was determined using plaque reduction neutralization test (PRNT). (**A**), Sera from mice immunized with VLP-DENV^1^ were diluted to 1:10 and tested against live DENV^1^ virions. (**B**), Sera from mice immunized with VLP-WNV^Kun^ (1:10 dilution) were tested against live WNV^Kun^ virions. Neutralization activity of sera was expressed as the percentage of virus neutralization, based on plaque determinations (average of three determinations, m ± SD).

***(ii) Induction of flavivirus neutralizing antibodies.*** The antiviral neutralization response was evaluated using the plaque reduction neutralization test **(**PRNT). PRNT is the method of choice for evaluating the potency and efficacy of anti-flavivirus vaccines, as it determines the flavivirus neutralization activity (NA) of a serum, i.e. the capacity of circulating antibodies to block virus infection. PRNT showed that sera from 3 out of 5 mice injected with VLP-DENV^1^ contained neutralizing antibodies against DENV^1^, with NA titers ranging from 20 to 30% at dilution 1:10 (Figure 9A). For mice immunized with VLP-WNV^Kun^, 3 sera out of 5 had neutralizing antibodies against WNV^Kun^ virus, with NA titers ranging from 25 to 50% at dilution 1:10 (Figure 9B). Although relatively modest, this neutralization response against DENV^1^ and WNV^Kun^ viruses was significant. No NA against either virus was detected in sera of mice immunized with VLP-0: the PRNT values obtained were within the limits of the background values (-10 to +10% NA) at all dilutions tested (not shown).

***(iii) Absence of correlation between DENV***^***1***^**and anti-WNV**^**Kun**^***antibody levels and virus neutralization activity*.** Comparison of the ELISA and PRNT data showed that for DENV^1^, mice #4 and #5 developed the highest levels of total anti-DIII-DENV^1^ antibodies (detected by ELISA; refer to Additional file 1: Figure S1), whereas sera of mice #2, #3 and #4 were the highest in NA titer (detected by PRNT; refer to Figure 9A). Only one animal, mouse #4, had both high levels of total anti-DIII-DENV^1^ antibodies and significant levels of DENV^1^ virus neutralizing antibodies. In the case of WNV^Kun^, mice #1 and #4 developed the highest levels of total anti-DIII-WNV^Kun^ antibodies, whereas sera of mice #2, #3 and #5 showed the highest NA against WNV^Kun^ virus (compare Figure 9B and Additional file 2: Figure S2). There was an apparent discrepancy between ELISA and PRNT: PRNT detected neutralizing antibodies in some of the immunized animals whereas their total antiviral antibodies were at background levels in ELISA. This was likely due to technical differences in the assays: in ELISA, live WNV^Kun^ and DENV^1^ virions were immobilized on a solid support, whereas in PRNT, they were dispersed in a liquid medium to allow for their interaction with antibodies. This allowed neutralizing antibodies to bind to cryptic epitopes while the virions “breathe” in solution during incubation, as recently suggested [65]. Such structural dynamics will not be possible in immobilized virions. Whatever the mechanism, the absence of correlation between the total levels of antibodies induced by VLP-DIII in immunized animals and their capacity to neutralize the live viruses implied that only a certain population of antibodies were neutralizing.

***(iv) Anti-Gag response***. The antibodies induced against the core components of the VLP carriers, were evaluated by ELISA, using recombinant Pr55GagHIV polyprotein expressed in AcMNPV-Pr55Gag^HIV^-infected Sf9 cells, with mock-infected Sf9 cell lysate as negative control. We observed significant levels of anti-Gag response in some of the immunized mice (Additional file 3: Figure S3). However, there was no correlation between the level of the anti-Gag response and that of the anti-DIII response whatever the immunogen used, VLP-0 versus VLP-DIII-DENV^1^ or VLP-DIII-WNV^Kun^ (compare Additional file 1: Figure S1, Additional file 2: Figure S2 and Additional file 3: S3). Likewise, there was no correlation between the level of anti-Gag antibodies and the level of DENV^1^ or WNV^Kun^ neutralizing antibodies (compare Figure 9 with Additional file 3: Figure S3). This indicated that the anti-flaviviral DIII response was independent of the response towards the VLP carriers, and was more complex than the simple reflection of the whole responsiveness of immunized animals.

## Discussion

In the present study, DENV serotype 1 was our Dengue virus prototype, and the extensively studied Kunjin strain was selected as our WNV prototype. WNV Kunjin, which was isolated in Australia, belongs to lineage 1, and strains in lineage 1 are generally more virulent and neuro-invasive. The choice of the strategy of VLP-display of the structural DIII domain of flavivirus envelope glycoprotein for potential vaccine antigen was based on the following considerations. The structure of DIII resembles that of the constant domain of immunoglobulins, and its flexible loops exposed on the exterior of the mature virions are the major determinants of the global immunogenicity of flaviviruses [66,67]. DIII has been shown to be highly immunogenic. More importantly, it carries neutralizing epitope(s) of the envelope glycoprotein, and thus is capable of inducing a robust immune response and neutralizing antibodies [5,26-37,56]. Antibodies binding to epitopes on DIII have been detected in sera of patients with DENV and WNV infections [68-72]. The presentation of solely the DIII domain, instead of the whole envelope glycoprotein, has the advantage of focusing the immune response on the neutralizing epitopes on DIII. Furthermore, this strategy excludes the DII domain, which harbours immunodominant, but non-neutralizing epitopes. Considering the over-representation of non-neutralizing, cross-reactive, domain II-binding antibodies in WNV-infected patients, DIII-based vaccines reduce the potential risk of antibody-dependent enhancement of infection in vaccinated individuals if infected with a heterologous flavivirus [73].

The expression of DIII proteins in insect cells was justified by the fact that the flavivirus life cycle involves a mosquito vector as an intermediate host. Thus, flaviviral proteins will follow a biosynthetic pathway and posttranslational modifications in Sf9 cells similar to that which naturally occur in the insect vector. VLPs, as other particulate structures, are known to possess adjuvant properties [5,23], and this represents a significant advantage for vaccination strategies. The choice of recombinant Pr55Gag^HIV^ as our proof-of-concept was determined by the high productivity of VLPs when recombinant HIV-1 *gag* gene was expressed in baculovirus-infected cells, compared to other systems [10]. We have previously shown that in AcMNPV-Pr55Gag^HIV^-infected Sf9 cells, 2 × 10^8^ to 5 × 10^8^ Pr55Gag^HIV^ molecules/cell were produced and released into the extracellular medium at 48 hpi. This corresponded to 5 × 10^4^ to 1 × 10^5^ VLPs per cell, if one considers that 5 × 10^3^ Gag molecules are required to form one VLP [51,52]. Interestingly, we found that pseudotyping of VLPs by DIII-RIgE was not restricted to the lentiviral Pr55Gag^HIV^ backbone, but was also observed with distantly related retroviral Gag polyproteins, such as MLV Gag polyprotein Pr67Gag^MLV^. This implied that our VLP-display platform could be extended to MLV-based VLPs, which has been clinically approved as vector for gene therapy by health regulatory agencies such as FDA.

We found that when the DIII domains of the envelope glycoproteins of DENV^1^ and WNV^Kun^ were fused to the N-terminal signal peptide (SP) of human CD16, and to the transmembrane and cytoplasmic domains of the gamma chain of the human high affinity receptor for IgE (RIgE) at their C-termini, the resulting fusion constructs DIII-DENV^1^-RIgE and DIII-WNV^Kun^-RIgE were addressed to the plasma membrane of human and insect cells. None of the fusion protein seemed to be glycosylated: the DIII domain did not carry any canonical glycosylation site, and DIII-DENV^1^-RIgE and DIII-WNV^Kun^-RIgE migrated as sharp bands in SDS-PAGE, with apparent molecular weights consistent with their amino acid compositions and theoretical molecular weight values.

When DIII-DENV^1^-RIgE and DIII-WNV^Kun^-RIgE were coexpressed with Pr55Gag^HIV^ in insect cells, they were efficiently incorporated into VLPs, and released into the extracellular medium as VLP-displayed DIII-RIgE molecules. Immunological analyses showed that the DIII domain of the two chimeric constructs were accessible at the surface of the VLPs. In addition, the ectodomain of DIII-DENV^1^-RIgE was immunologically reactive against specific patient antibodies. This demonstrated the functionality of using the VLPs to present structural domains of the flavivirus envelope glycoprotein as a substitute to virus-based vaccines.

The results of mice immunization showed that VLPs pseudotyped with our DIII-RIgE construct represented a promising platform for flavivirus vaccines, since a neutralization response against DENV and WNV was induced in a significant number of mice (60%). In one case for each vaccine antigen prototype, the NA titer reached 50% at 1:10 serum dilution, a value which fulfilled the WHO criterion for sera to be considered as neutralizing (http://whqlibdoc.who.int/hq/2007/who_ivb_07.07_eng.pdf). Our previous immunization regimen, which used bacterially expressed DIII and GpC adjuvant administered intraperitoneally to mice, resulted in 80-90% virus neutralization effect at 1:128 to 1:16 serum dilutions [29]. However, this neutralization titer was obtained with 100 μg recombinant DIII protein per vaccine dose, i.e. a 250- to 500-fold higher amount compared to our VLP-displayed DIII-RIgE.

The unexpected absence of correlation between anti-DENV^1^ and anti-WNV^Kun^ antibody levels in our ELISA and PRNT data might be attributed to the suboptimal antibody response induced by our prototype pseudotyped VLP vaccines. Optimisation of our pseudotyping process to further increase the copy number of DIII-IgE per VLP, and our immunization protocol (e.g. immunogen dosage, number of boosts, etc.) has been planned for as part of our future work to fulfill the WHO criterion for neutralizing sera in all immunized animals. However, the fact that the injection of VLPs carrying a limited domain of the flavivirus envelope glycoprotein was sufficient to induce neutralizing antibodies in animals confirmed previous observations, made by our laboratory and others, that DIII carried a major neutralizing epitope (reviewed in [74]). Neutralization of flaviviruses by polyclonal antibodies has been shown to result from a complex interplay between several viral components and host factors [74]. More recently, a high-resolution crystallographic analysis of DENV DIII in complex with a mouse monoclonal antibody capable of neutralizing all four DENV serotypes has dissected the molecular determinants of inter-DENV cross-reactivity [75]. Generation of VLPs pseudotyped with the DIII-RIgE of DENV serotypes 2, 3 and 4 have also been planned for, to allow for comparison of cross-neutralization activities between the sera raised through immunization with each DIII-RIgE and different DENV serotypes.

In conclusion, our data showcased, to the best of our knowledge, the innovative use of pseudotyped Gag VLPs as a vaccine vector for the display of flaviviral antigens. Although our results were relatively modest for our first two prototypes, DENV^1^ and WNV^Kun^ neutralizing antibodies were indeed induced. With further optimization in our future works, we are confident that our VLP-displayed CD16-RIgE-based platform will be developed into a versatile vaccine vector against different flaviviruses and other viral pathogens.

## Competing interests

The authors declare that they have no competing interests.

## Authors contributions

Conceived and designed the experiments: SSH PB MLN. Performed the experiments: AJSC MLCT CV GG SSH. Analyzed the data: AJSC MLCT CV GG PB MLN SSH. Wrote the paper: PB AJSC SSH MLN. All authors approved the final manuscript.

## Supplementary Material

Additional file 1: Figure S1 Induction of antibodies against DENV^1^ virions in mice immunized with VLP-DENV^1^ versus control, nonpseudotyped VLP-0. Serial dilutions of each mouse serum were tested against DENV^1^ virions adsorbed on ELISA wells. Data shown are the average of three determinations (m ± SEM). The different symbols refer to sera from individual animals. Click here for file

Additional file 2: Figure S2 Induction of antibodies against WNV^Kun^ virions in mice immunized with VLP-WNV^Kun^, versus control, nonpseudotyped VLP-0. Serial dilutions of each mouse serum were tested against WNV^Kun^ virions adsorbed on ELISA wells. Data shown are the average of three determinations (m ± SEM). The different symbols refer to sera from individual animals. Click here for file

Additional file 3: Figure S3 Antibody response against the VLP core component. Aliquots of lysates from mock-infected or AcMNPV-Pr55Gag^HIV^-infected Sf9 cells containing recombinant Pr55Gag polyprotein, were coated on ELISA wells. Wells were reacted with sera (1:10 dilution) of mice immunized with control, nonpseudotyped VLP-0 (light grey bars), VLP-DENV^1^ (hatched bars), or VLP-WNV^Kun^ (black bars). Data shown are the average of three determinations (m ± SEM), after subtraction of the background value given by mock-infected Sf9 cell lysates. Click here for file
