# Supplementary figures and images for: A novel platform for virus-like particle-display of flaviviral envelope domain III: induction of Dengue and West Nile virus neutralizing antibodies
Source: Virol J. 2013 Apr 24;10:129. doi: 10.1186/1743-422X-10-129 (PMC3668303; doi:10.1186/1743-422X-10-129)

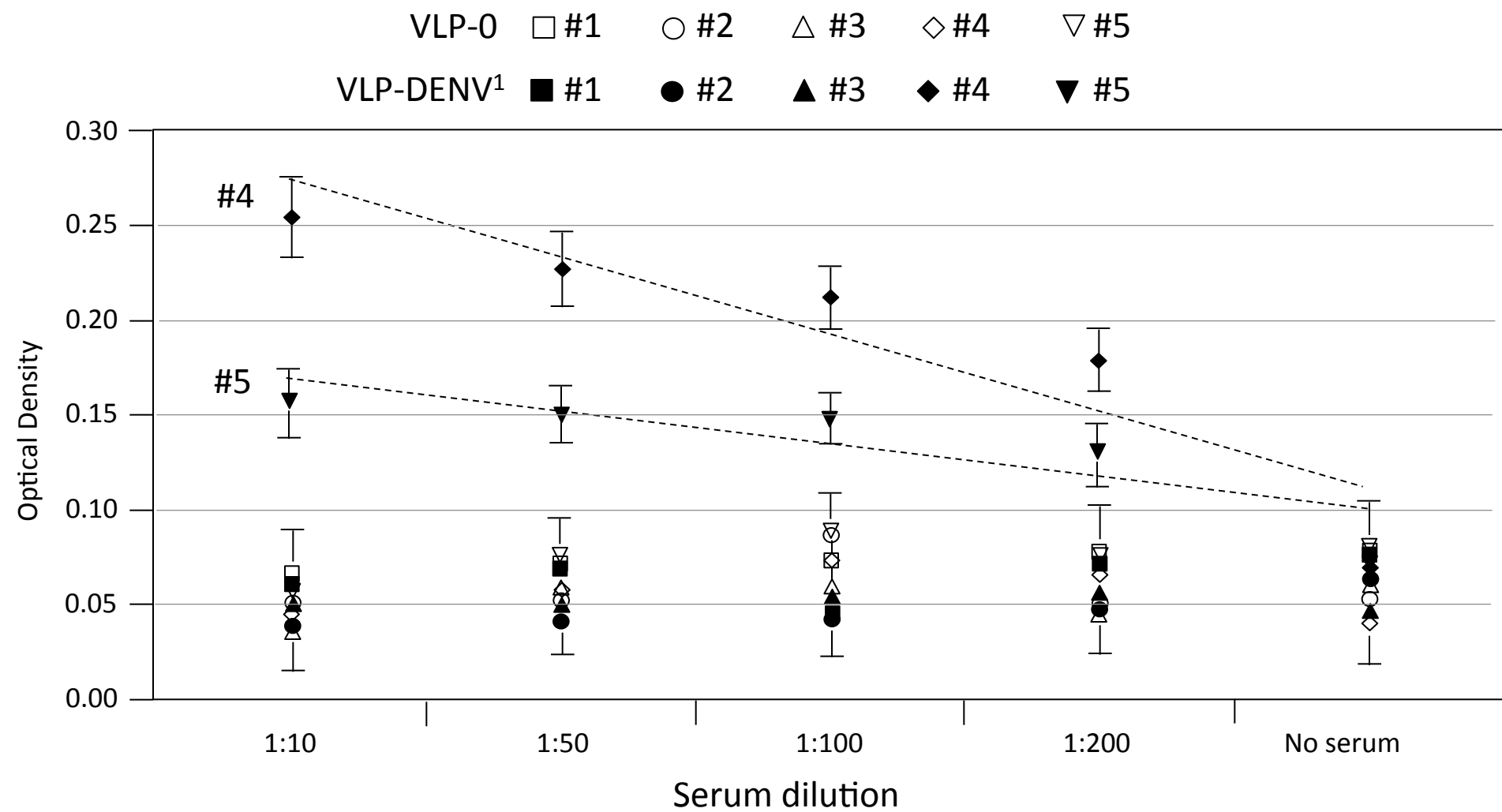

Supplementary Figure S1

Supplement: Additional file 1: Figure S1 — Induction of antibodies against DENV1 virions in mice immunized with VLP-DENV1 versus control, nonpseudotyped VLP-0. Serial dilutions of each mouse serum were tested against DENV1 virions adsorbed on ELISA wells. Data shown are the average of three determinations (m ± SEM). The different symbols refer to sera from individual animals. [file 1743-422X-10-129-S1.pdf]

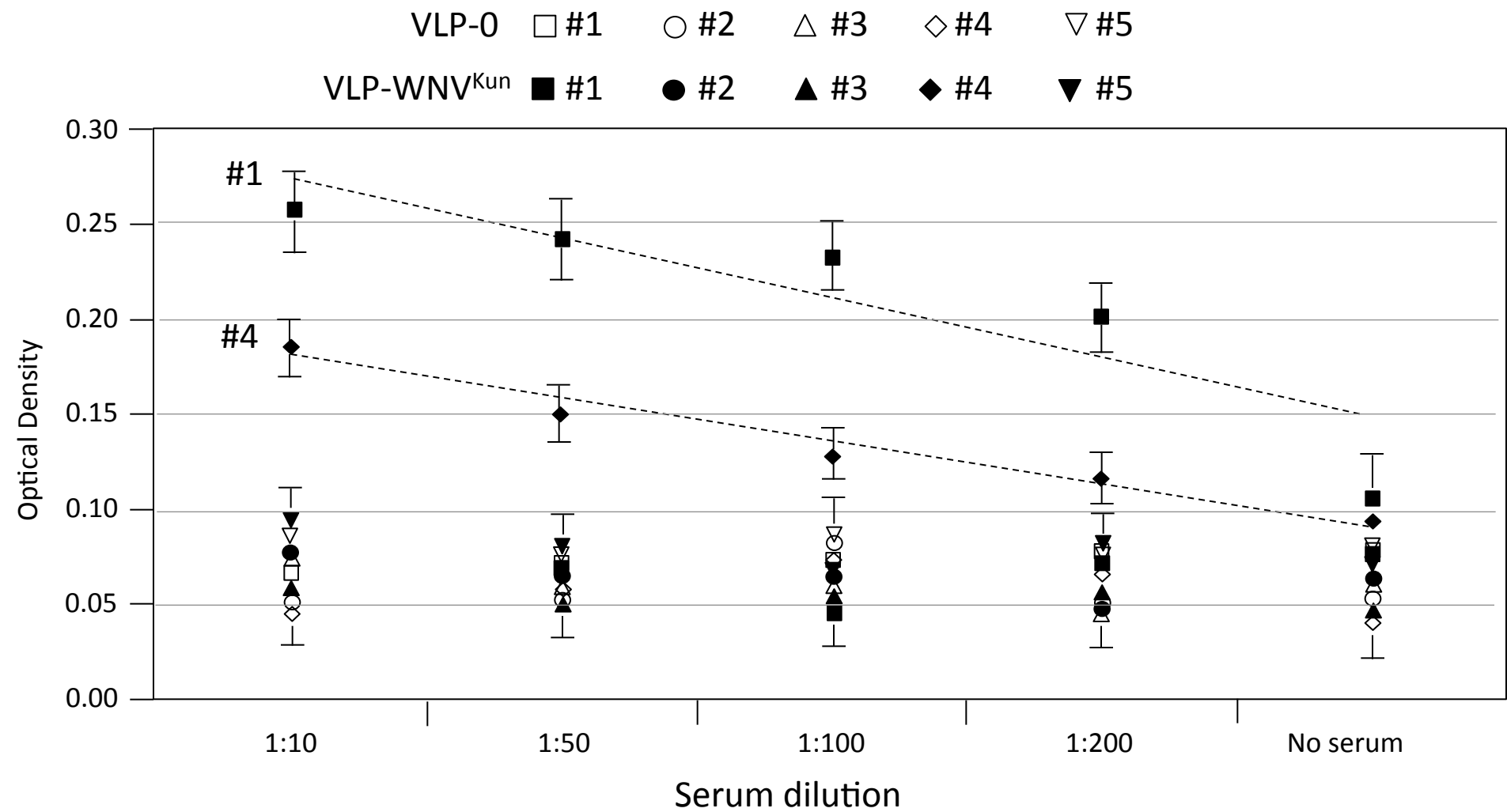

Supplementary Figure S2

Supplement: Additional file 2: Figure S2 — Induction of antibodies against WNVKun virions in mice immunized with VLP-WNVKun, versus control, nonpseudotyped VLP-0. Serial dilutions of each mouse serum were tested against WNVKun virions adsorbed on ELISA wells. Data shown are the average of three determinations (m ± SEM). The different symbols refer to sera from individual animals. [file 1743-422X-10-129-S2.pdf]

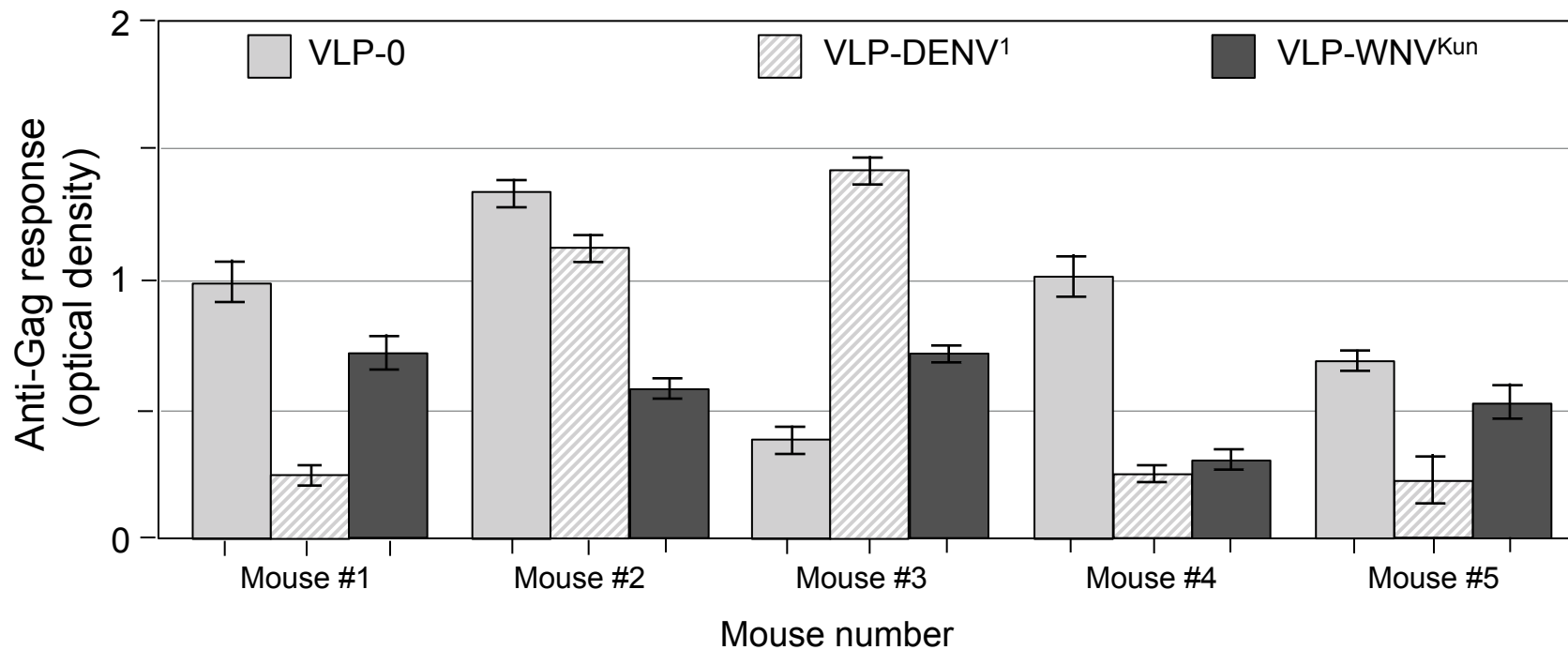

Supplementary Figure S3

Supplement: Additional file 3: Figure S3 — Antibody response against the VLP core component. Aliquots of lysates from mock-infected or AcMNPV-Pr55GagHIV-infected Sf9 cells containing recombinant Pr55Gag polyprotein, were coated on ELISA wells. Wells were reacted with sera (1:10 dilution) of mice immunized with control, nonpseudotyped VLP-0 (light grey bars), VLP-DENV1 (hatched bars), or VLP-WNVKun (black bars). Data shown are the average of three determinations (m ± SEM), after subtraction of the background value given by mock-infected Sf9 cell lysates. [file 1743-422X-10-129-S3.pdf]
